# Antisense oligonucleotides as a precision therapy for developmental and epileptic encephalopathies

**DOI:** 10.1111/cns.70050

**Published:** 2024-11-11

**Authors:** Paloma García Quilón, Greta Volpedo, Serena Cappato, Loretta Ferrera, Federico Zara, Renata Bocciardi, Antonella Riva, Pasquale Striano

**Affiliations:** ^1^ Department of Neurosciences, Rehabilitation, Ophthalmology, Genetics, Maternal and Child Health (DINOGMI) University of Genoa Genoa Italy; ^2^ Unit of Medical Genetics IRCCS Istituto Giannina Gaslini Genoa Italy; ^3^ Pediatric Neurology and Muscular Diseases Unit IRCCS Istituto Giannina Gaslini Genoa Italy

**Keywords:** antisense oligonucleotides, clinical studies, DEE, epilepsy, gene therapy, preclinical studies

## Abstract

Developmental and epileptic encephalopathies (DEEs) comprise a complex spectrum of neurological disorders characterized by neurodevelopmental delay and early‐onset seizures primarily caused by diverse genetic mutations. Traditional treatments have largely been symptomatic, focusing on seizure control without addressing the underlying genetic causes. The advent of gene therapy, particularly through antisense oligonucleotides (ASOs), offers a promising avenue toward targeted therapeutic interventions. ASOs by virtue of their ability to modulate gene expression at the mRNA level represent a sophisticated approach to counteract the effects of pathogenic mutations. This review delves into the recent advancements in ASO technology, highlighting its application in preclinical and clinical settings for DEEs. We present evidence of the efficacy of ASOs in ameliorating disease phenotypes in vitro and in vivo, alongside promising outcomes from ongoing clinical trials. The therapeutic landscape for DEEs is on the cusp of significant transformation, underscored by the potential of ASOs to offer precise, personalized, treatments that extend beyond symptomatic relief to potentially rectify the genetic underpinnings of these disorders.

## Graphical abstract

ASOs have led to important advancements in mRNA regulation for treating developmental and epileptic encephalopathies (DEEs). The mechanisms of action may vary: RNA function blocking could be obtained by modulating alternative splicing or through steric hindrance; similarly, RNA degradation could be obtained through RNase H1. However, upregulation of the final haploinsufficient protein product can also be achieved. Pre and clinical studies pave the way for potential innovative therapies.

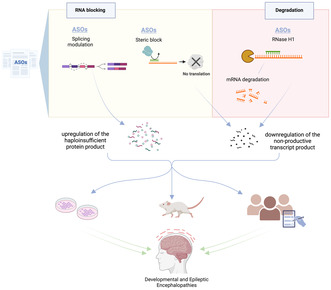

## 1 INTRODUCTION

Developmental and epileptic encephalopathies (DEEs) are a heterogeneous group of neurological disorders brought together by the invariable presence of developmental impairment and frequent seizures.^1^ It was previously thought that seizures themselves or the epileptiform activity on the electroencephalogram (EEG) could impact the normal developmental trajectories; although, recently, the term “developmental encephalopathy” was added to underline that the development itself is already compromised as a result of altered neuronal sprouting/synaptic plasticity and neural networks building.^2^ Single‐gene (called monogenic) causes have been found in about one‐third of the cases and mutations in voltage‐gated neuronal channels, including sodium and potassium channel genes, account for about 2% of the total.^3^ Within these, major causative genes for DEEs include *SCN1A*, *SCN2A*, *SCN8A*, and *KCNT1*. *SCN1A* (#OMIM 182389), encoding for the α‐subunit of the voltage‐gated sodium channel Na_V_1.1, was first associated with Dravet syndrome (DS, #OMIM 607208) in the 2000s.^4^ The prevalent mechanism is a loss of the channel function, leading to reduced activity of inhibitory GABAergic interneurons and an overall increase in neuronal excitability.^4^, ^5^ However, also gain‐of‐function (GoF) mutations in *SCN1A*, historically associated with familial hemiplegic migraine type 3, have recently been associated with encephalopathic phenotypes, including neonatal developmental and epileptic encephalopathy with movement disorder and arthrogryposis, and early infantile DEEs with/without movement disorder.^5^, ^6^ *SCN2A* (#OMIM 182390) encodes for the Na_V_1.2 sodium channel. GoF pathogenic variants in *SCN2A* often lead to early infantile DEEs, whereas loss‐of‐function (LoF) generally causes later‐onset epilepsy with a moderate impact on executive functions or autism spectrum disorder (ASD) without epilepsy.^7^ Another relevant sodium channel gene is *SCN8A* (#OMIM 600702), which encodes for the α‐8 sodium channel subunit (Na_V_1.6). The observed range of phenotypes indicates that GoF mutations are responsible for DEEs, including DS‐like phenotypes.^5^, ^8^ With distinct pathogenic mechanisms, GoF mutations in the potassium channel *KCNT1* (#OMIM 608167) also play a critical role in early‐onset DEEs.^9^

Pathogenic variants in genes “other” than neuronal channels, such as those building synaptic proteins and receptors, are also known to cause DEEs. Rett syndrome (RS) is a neurodevelopmental disorder caused in 95% of the cases by mutations in *MECP2* located on the X chromosome, hence primarily affecting females. The mutation in *MECP2* determines the disruption of the Methyl CpG binding protein 2 (MeCP2), which is essential for regulating gene expression in the brain by modifying chromatin assembly.^10^ The resulting phenotype is that of RS with normal neurodevelopment during the first few months of life, invariably followed by a progressive loss of motor and language skills, as well as the onset of stereotypic hand movements, growth difficulties, and sleep disturbances. Affected girls may also experience seizures, coordination problems, and autism‐related symptoms. Overall the syndrome leads to significant functional impairment, severely affecting the quality of life and reducing life expectancy, primarily due to respiratory and cardiorespiratory complications.^11^

However, despite strong epidemiological evidence of a genetic basis, there is still a long way to identify specific drug targets as most treatments only focus on symptomatic seizure control and real etiological treatment is still in its infancy.^12^ Particularly, RNA‐based targeted therapies have recently gained more attention following the success obtained in some preclinical and early clinical works; within these, antisense oligonucleotides (ASOs) have shown the greatest impact and have been recognized as one of the most advanced RNA‐based therapeutic modalities, reinforced by successful preclinical and early clinical trials in diverse neurological disorders including spinal muscular atrophy and Duchenne muscular dystrophy.^13^ The main advantage of ASOs as a therapeutic approach lies in their high specificity of interaction with the target, thus outperforming traditional small molecule‐based therapies.^6^ For this reason, these therapies are starting to be used for the treatment of DEEs.^1^, ^14^

**FIGURE 1:**
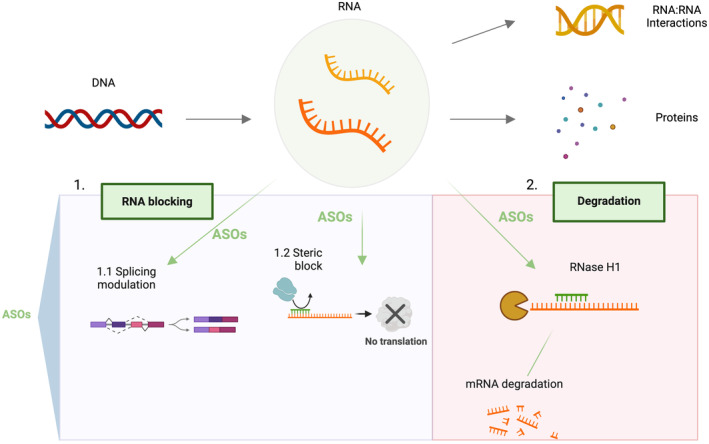
Mechanism of action of ASOs on target RNAs. In the upper panel of the figure is schematically represented the flow of the genetic information leading to gene expression and protein synthesis. DNA can be transcribed in different types of RNA, here noncoding, and protein‐coding RNAs are represented. Noncoding RNAs play crucial roles in the regulation of gene expression through specific interactions and mechanisms. ASO may be applied to target specific RNAs thereby modulating gene or allele‐specific expression, by splicing modulation; masking of specific mRNA sequences to affect translation; targeting RNA for degradation. Created with biorender.com.

ASOs work by specifically targeting the RNA of interest by Watson‐Crick base pairing, thus regulating gene expression at post transcriptional level through different mechanisms, schematically represented in Figure 1, involving (1) RNA blocking, such as (1.1) modulation of splicing, to include or exclude specific introns/exons thus promoting the expression of productive mRNA; (1.2) regulation of the translation efficiency, for example, by masking specific sequences to block or decrease translation; or (2) targeting coding or noncoding RNAs (such as microRNAs) for degradation. In this case, gene silencing is largely achieved by mRNA degradation through the recruitment of RNase H1. Particularly, upon binding of the ASO to the target mRNA, an RNA‐ASO hybrid is formed which induces enzymatic degradation by RNase H1, thereby reducing mRNA levels.^15^, ^16^

Focusing on the fine biological aspects, ASOs are designed to target various mRNA regions of the different genes. They are selected for their transcription regulation abilities, their in vivo activity, and their tolerability. They must also have a complementary pre‐mRNA sequence, 100% identity between mouse and human, and no significant cross‐hybridization with any other genes in the mouse or human genomes. Because both GoF and LoF variants of the same gene could be pathogenic, it is crucial to identify the mechanism of action of each variant to specifically design the ASO therapy. LoF alleles are typically associated with haploinsufficiency, where a protein level of 50%, derived from the unaffected allele, is insufficient to prevent phenotypic manifestations.^18^ In these cases Targeted Augmentation of Nuclear Gene Output (TANGO)‐based ASO treatment has shown to be effective by regulating protein synthesis of the remaining normal copy of the damaged gene. Although, the mechanism could also be the opposite and ASOs could reduce the mRNA transcript of some GoF mutations.^2^ For example, the ASO sequence designed could be identical to the genomic sequence of the 3′ untranslated region (UTR) to the end of targeting it and reducing the resultant transcript abundance.^19^

In this review paper, we will discuss the application of ASOs treatment in major DEEs.

## 2 METHODS

**FIGURE 2:**
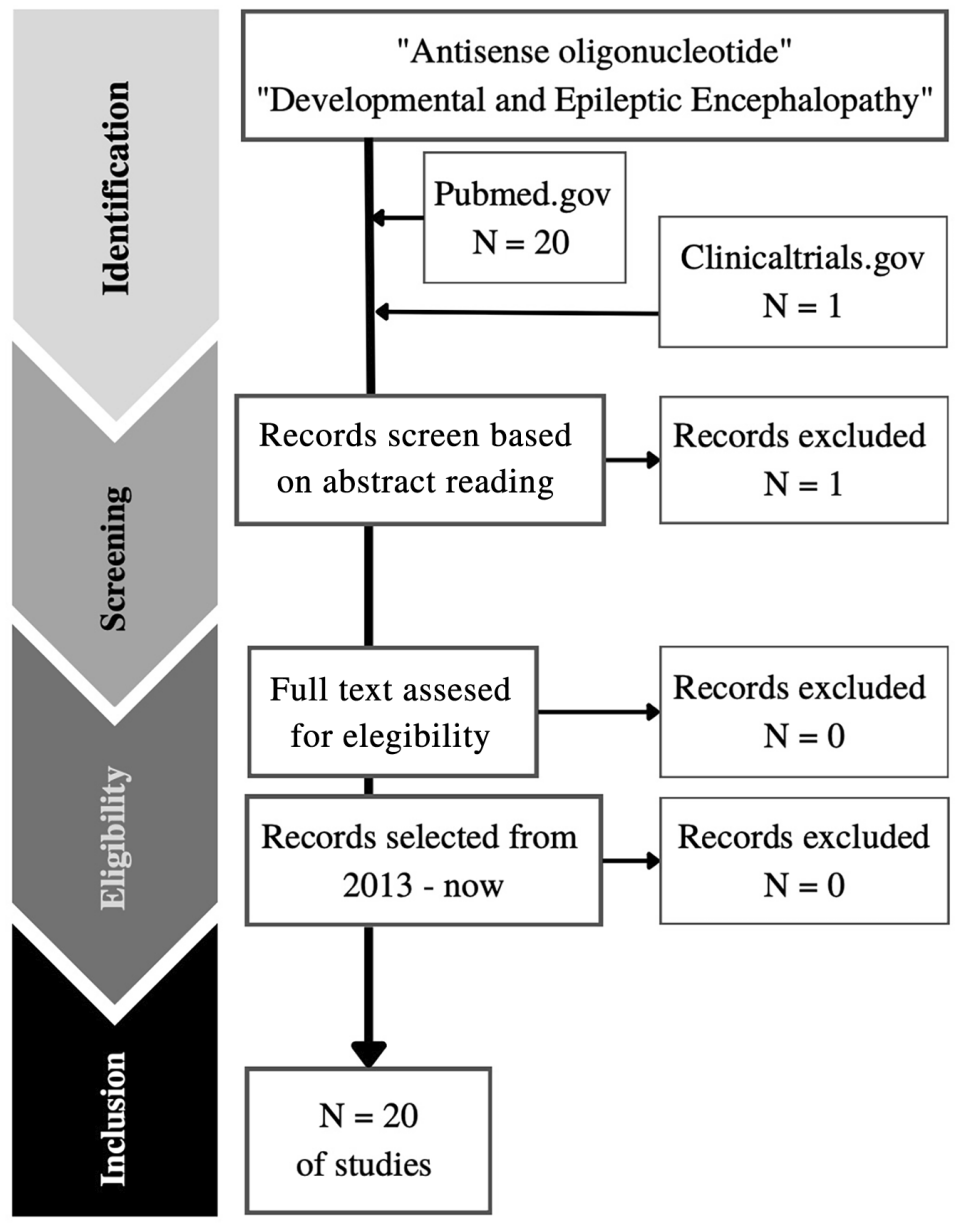
Schematic diagram on the selection of papers for the body of the review.

To identify relevant articles, a systematic search was performed on PubMed and clinicaltrials.gov databases using the search terms “Antisense oligonucleotide” AND “developmental and epileptic encephalopathies.” The search spanned from January 2010 to October 2023. Duplicate records and articles not in English were excluded during the initial screening based on title and abstract content. Then, a second reviewer conducted a comprehensive evaluation of the full texts to determine eligibility. In total, we analyzed 20 papers, encompassing studies on the efficacy and safety of ASOs through *in vitro* and *in vivo* settings, as well as in early phase clinical trials (Figure 2).

## 3 PRECLINICAL STUDIES

One of the principal aspects that can be regulated through targeted ASO treatment is the function of the poison exons (PEs). The PEs are noncoding exons that undergo alternative splicing, which can affect the final amount of protein translated. Usually, PEs are small (<100 bp) and conserved exons containing an *in‐frame* stop codon; when this premature truncation codon is incorporated into a coding region, it triggers degradation of the mRNA through nonsense‐mediated decay (NMD). Therefore, protein levels may be reduced as the inclusion of PEs in a transcript mediates unproductive splicing. Biologically, the function of PEs is undefined, but they likely regulate the amount of functional protein within cells in a more agile way than can be achieved by activating and silencing transcription.^20^ These mechanisms are important in neuronal development and alternative splicing of these exons is tightly autoregulated. Certain PEs occur in epilepsy‐associated genes, in particular in the DEEs. Specifically, some PEs have been identified in patients with DS, such as the 20N PE, whose presence results in an increased inclusion of 20N in a minigene assay, leading to NMD in the neurons of affected individuals, and, essentially, *SCN1A* haploinsufficiency.^20^

Blocking PE inclusion leads to increased production of full‐length transcripts of both alleles, even though one of them may be nonfunctional. Experiments to test the ability of an ASO to avoid PEs inclusion and restore the physiological protein levels have largely been performed in *in vitro* models, whereas tests are currently limited in *in vivo* models.^12^ To analyze mutations in sodium channels, various researchers^1^, ^2^, ^17^ have used C57BL/6J mice to study genes including *SCN1A*, *SCN2A*, and *SCN8A*.

Particularly, Han et al.^17^ analyzed the effect of a specifically designed ASO‐22 targeting the 20N PE and the surrounding intronic sequences of the human *SCN1A* gene, whose *de novo* mutation results in haploinsufficiency of the α‐1 subunit of the voltage‐gated sodium channel and leads to DS. The authors co‐cultured human neural progenitor cells (ReNcells) and the ASO‐22, which was internalized by free uptake. The *SCN1A* expression was found to increase in cultured cells by the analysis of productive and nonproductive *SCN1A* transcripts using RT‐PCR and qPCR to detect the 20N exon. According to these results, ASO‐22 potently and specifically increases *SCN1A* mRNA in human neural progenitor cells.

Within the study of PEs, the 20N exon of *SCN1A* is the human homologous of the 21N exon in mice. Han et al.^17^ used a well‐recognized model of the DS phenotype. For this, the exon 1 of *Scn1a* was deleted in the *Scn1a* tm1Kea mouse model, resulting in haploinsufficiency with decreased levels of Na_V_1.1. The final cohort of mice was maintained by breeding heterozygous animals to 129S6/SvEvTac mice, resulting in 129S6.*Scn1a* ^+/−^ model. For all experiments, C57BL/6J mice were crossed with 129S6.*Scn1a* ^+/−^ mice to generate the first generation of offspring. These mice typically have a 50% rate of premature mortality due to sudden unexpected death in epilepsy (SUDEP). The use of the ASO‐22 treatment to target the nonsense‐meditated decay of *Scn1a* mRNA in this model resulted in a significant increase in the survival of DS mice, by more than four times. As secondary endpoints, the exposure, target engagement, and Na_V_1.1 expression in the brains of ASO‐treated and PBS (phosphate‐buffered saline)‐treated DS mice were compared. After 14 weeks of injection, the expression of Na_V_1.1 protein in the brains of ASO‐treated DS mice increased to levels comparable to those of wild‐type (WT) brains, while this was not seen in PBS‐treated mice.^17^ Then, P2 wild‐type mice received a single ASO‐22 ICV injection and brain tissue was collected 5 days later and examined to assess variations in the expression of both nonproductive and productive Scn1a transcripts, as well as the levels of Na_V_1.1 protein. The results showed an increase in productive Scn1a mRNA transcripts and a decrease in nonproductive transcripts, as well as an increase in Na_V_1.1 abundance.^17^ Given these results, post natal day 2 (PD2) mice were treated with ASO‐22 by a single ICV injection, and a 90‐day survival analysis showed 97% survival of Scn1a tm1Kea mice treated with ASO. Furthermore, at 7 and 14 weeks after injection, ASO‐22 levels were still present at concentrations sufficient to produce an increase in the *Scn1a* transcript levels and a Na_V_1.1 protein abundance similar to WT levels. Finally, EEG data showed that Scn1a tm1Kea mice treated with ASO‐22 experienced fewer seizures and the latency to the first seizure was prolonged.^17^

A seminal study was previously conducted by Hsiao et al.^19^ providing evidence of the *in vitro* upregulation of *SCN1A* using an AntagoNAT antisense RNA, targeting different regions of antisense noncoding RNA (SCN1ANAT). The specific ASO was transfected into various cell lines (i.e., SK‐N‐AS and HepG2 from humans, and 3T3 and Neuro2A from mice), in which an increase in *SCN1A* mRNA levels was obtained after treatment. Then, the knock‐in Scn1aE1099X mouse model of DS was also studied. This model was created by introducing a targeting vector containing a modified version of the *Scn1a* gene. To this end, 15 kb mouse genomic DNA fragment including exons 10–17 of *Scn1a* was inserted into a cloning vector, and in exon 17 the codon encoding glutamic acid at position 1099 was mutated to a stop codon (TAG) using a PCR method, resulting in the E1099X variant that disrupts protein production. The specific antagoNATs (CUR1901) were injected intrathecally (IT) in 7‐week‐old mice. As a result, the authors observed an improvement in the disease phenotype with a decrease in seizure frequency and severity. Moreover, they calculated that an increase in brain levels of *Scn1a* was directly associated with a dose‐dependent reduction in seizure frequency, as well as heat‐induced seizures. Finally, an improvement in the electrophysiological conditions of parvalbumin‐positive (PV) neurons (GABAergic interneurons), whose defect is responsible for DS, was also observed. In that same study, tests were performed to target the human SCN1ANAT with AntagoNATs; the Vero76 African green monkey model, whose SCN1ANAT is highly homologous to humans, was used as a test subject. The AntagoNATs were injected IT and, in this model, an increase in brain levels of *Scn1a* mRNA was also obtained.^19^

Another mouse model heterozygous for *Scn1a* ^+/−^ was treated with STK‐001 (a specific ASO for *SCN1A*). STK‐001 treatment rescued the reduced intrinsic excitability of PV inhibitory interneurons associated with this syndrome and reduced seizures, prolonging survival in the model.^21^ Together, these studies provide preclinical evidence that ICV administration of TANGO‐based ASO increases the production of the *Scn1a* mRNA transcripts and Na_V_1.1 protein in *Scn1a* ^+/−^ mouse brains, extending the survival of *Scn1a* haploinsufficient mouse models. As a matter of fact, the first patients were recently enrolled in a clinical trial to investigate the efficacy, safety, and pharmacodynamics of STK‐001 (also called ASO‐22) administration in humans.^22^

Lenk et al.^3^ cultured primary cortical neurons from C57BL/6J E14 embryos for 3 days with different concentrations of *Scn8a* ASO. Reducing transcript levels is a reasonable therapeutic strategy, considering that the pathogenic mechanism of SCN8A encephalopathy involves neuronal hyperexcitability due to GoF mutations. ASOs hybridize to mRNAs by Watson‐Crick base pairing, resulting in ribonuclease H degradation, translation inhibition, or impaired splicing. Then, the *Scn8a* transcripts in neurons were quantified using TaqMan gene expression assay. The results showed that ASO treatment could reduce *Scn8a* transcript abundance in a dose‐dependent trend, proving the potential for the treatment of *SCN8A*‐related epilepsy.^3^

*In vivo* studies were also performed by Lenk et al.^3^ using a mutant mouse model with the conditional *Scn8a* allele (Scn8acond) activated by the Cre recombinase to specifically express the p.R1872W pathogenic GoF variant. In this mutant mouse model at postnatal days 14–16 animals present with onset of seizures and die within 24 hours. The abundance of the *Scn8a* transcript (mRNA) in the brain was measured in WT mice treated with ICV injection of ASO. Control ASO did not reduce the *Scn8a* transcript, but a dose‐dependent reduction of *Scn8a* mRNA was obtained in *Scn8a* ASO‐treated mice. Given that deleting the *Scn8a* transcript is lethal in mice, a carefully adjusted dose of ASO was administered by ICV injection at PD2 to partially reduce *Scn8a* mRNA while maintaining sufficient levels to observe a therapeutic effect. The treatment delayed seizure onset and prolonged the survival of mutant mice expressing the pathogenic SCN8A R1872W^−/+^ mutation. The prolonged period of protection after a single injection suggests that ASO administration during a critical period of postnatal development could give long‐term seizure control also in patients. Interestingly, the ASO could also extend survival in a *Scn1a* ^+/−^ haploinsufficient mouse model of DS. Future testing of *Scn8a* ASO in other seizure models will be of great interest.^3^

For the study of *SCN2A*, an ASO gapmer was designed to target mice heterozygous for *SCN2A*: p.1883Q pathogenic variant, which mimics the human GoF phenotype with severe and early‐onset seizures, is equivalent to the human pathogenic variant p.R1882Q (Q/+).^2^ In this study, the *Scn2a* ASO was ICV injected and specifically designed to test the hypothesis that reducing Scn2a expression would be therapeutic for mice expressing the pathogenic GoF variant. As a result, ASO administration rescued the *Scn2a* premature death and seizure phenotype in mice. Finally, repeated administration of *Scn2a* ASO further extended survival, suggesting that repeated dosing in later development maintained the therapeutic level of *Scn2a* ASO, avoiding disease recurrence.^2^, ^23^ Based on these exciting results human trials are being planned.^5^

DEEs may be associated also with GoF mutations in potassium channel genes. Studies on potassium channels dealt with the *Kcnt1* mutation; specifically, the efficacy of a *Kcnt1* ASO was evaluated in a homozygous mouse model of a pathogenic *KCNT1*‐DEE variant exhibiting spontaneous seizures, behavioral abnormalities, and early death. Following a single ICV of the *Kcnt1* ASO at PD40, mice showed a nearly 90% reduction in *Kcnt1* mRNA levels, resulting in near‐complete abolition of seizures, prolonged survival, and improved performance on behavioral tests.^12^

To move forward, the *MECP2* duplication syndrome (featuring epilepsy, developmental delay, and premature death) has also been studied.^11^ Particularly, lymphoblastoid cells from patients with *MECP2* duplication and an ASO specifically designed to target multiple regions of the human *MECP2* pre‐mRNA were tested. Lymphoblastoid cells from the affected patients and age‐matched control donors were incubated with 4.8 μM control‐ASO. RNA was extracted from the lymphoblasts 48 h following transfection, and the experiment demonstrated a correction of *MECP2 *levels in a dose‐dependent manner. Then, similar studies were also conducted in animal models of the disorder. For their study Sztainberg et al.^11^ generated a conditional‐overexpressing Mecp2 mouse on a pure FVB/N model (Friend Virus B NIH Jackson), then they synthesized a targeting ASO to try to correct the abnormal expression levels. Transgenic *MECP2* duplication mice (MECP2‐TG) were treated with ASO and showed restoration of the abnormal EEG discharges and a clinical correlate of absent seizures. For detailed information, see Tables 1 and 2.

**TABLE 1:** *In vitro* studies of ASOs in DEEs.

Authors, year	Type of cells	Type of developmental encephalopathies	Primary endpoints	Outcomes
Sztainberg et al., 2015	Patient lymphoblastoid cells	*MECP2* duplication syndrome	Effects of ASO by mRNA and protein levels	ASO provokes a correction of MECP2 levels in a dose‐dependent manner
Hsiao et al., 2016	Human: SK‐N‐AS and HepG2 Mice: 3T3 and Neuro2A African green monkey: vero76	DS	Effects of ASO by mRNA and protein levels	ASO increase *SCN1A* mRNA levels after treatment
Han et al., 2020	ReNcells	DS	Effects of ASO by mRNA and protein levels	ASO increases productive *SCN1A* mRNA in human neural progenitor cells
Lenk et al., 2020	Primary cortical neurons from E14 embryos of strain C57BL/6J	DS	Effects of ASO by mRNA and protein levels	ASO produces a dose‐dependent reduction in transcript abundance

Abbreviations: ASO, antisense oligonucleotides; DEE, developmental and epileptic encephalopathy; DS, Dravet syndrome; ReNcells, human neural progenitor cells.

**TABLE 2:** *In vivo* studies of ASOs in DEEs.

Authors, year	Animal model of mutation	Methods	Type of developmental encephalopathies	Primary endpoints	Outcomes
Sztainberg et al., 2015	MECP2‐TG	ICV injection	*MECP2* duplication syndrome	To test whether upregulating Scn1a with ASO after birth would improve disease symptoms in vivo	ASOs successfully got rid of the abnormal EEG discharges, put an end to the behavioral seizures, and eliminated the electrographic seizure spikes
Hsiao et al., 2016	Scn1aE1099X	Injected IT	DS mouse model	*Scn1a* mRNA levels	An increase in brain levels of *Scn1a* mRNA was also obtained
Lenk et al., 2020	*Scn8a* cond	*Scn8a* ASO ICV injection at postnatal day 2	This model shows early onset of seizures, rapid progression, and 100% penetrance	Registration of seizures onset and survival	Dose‐dependent increase in length of survival in the mutated mice treated with ASO A single treatment with *Scn8a* ASO extended survival of DS mice
Han et al., 2020	*Scn1a* tm1Kea	ICV administration of a lead ASO	DS	Registration of SUDEP incidence in DS mice and NaV1.1 expression	ASO treatment resulted in greater than fourfold improvement in survival of the DS mice. The expression of Na_V_1.1 protein increased in ASO‐treated DS mouse brains to amounts indistinguishable from WT brains. Increase survival, decrease fewer seizures and latency to the first seizure was prolonged
Li et al., 2021	Male *Scn2a* Q/+ Gain‐of‐function	*Scn2a* ASOs ICV injection	Na channelopathies, equivalent to human epilepsy encephalopathy variant	*Scn2a* mRNA and protein levels	*Scn2a* ASO reduces mRNA and protein levels, rescues premature death and seizure phenotype in mice
Wengert et al., 2022	*Scn1a* ^+/−^	STK‐001	DS mouse model	Registration of seizures, survival, and excitability of cells affected by DS	Reduces seizures, prolongs survival, and rescues PV+ interneuron excitability
Burbano et al., 2022	*Kcnt1* ^−/−^	*Kcnt1* ASO ICV injection at postnatal day 40	Sodium‐activated potassium channel protein KNa1.1 channelopathies, produce a gain‐of‐function epileptic syndrome	Registration of seizures frequency, behavior abnormalities, and survival duration	*Kcnt1* ASO reduces seizures frequency, improves behavior abnormalities, and survival extends

Abbreviations: ASO, antisense oligonucleotides; DEE, developmental and epileptic encephalopathy; DS, Dravet syndrome; ICV, Intracerebroventricular injection; IT, intrathecal; SUDEP, sudden unexpected death in epilepsy.

## 4 CLINICAL STUDIES

Clinical trials are an essential part of testing the real effects of ASO‐based therapies in patients.

In recent years, several FDA‐ASOs have been approved for clinical use in neurodegenerative conditions such as spinal muscular atrophy (SMA), and Duchenne muscular dystrophy. Although, despite notable successes, they have faced several challenges; for example, in Huntington's disease, the trial failed to demonstrate significant clinical benefits, resulting in inconclusive outcomes.^24^, ^25^

A multicenter, randomized, double‐blind, placebo‐controlled Phase 1b/2a study was conducted to evaluate the safety, tolerability, pharmacokinetic, and pharmacodynamic profiles of single and multiple doses of WVE‐120101 and WVE‐120102 injected directly into the spinal canal. These ASOs target specific polymorphisms of the mutant mRNA of the *Huntingtin* (*HTT*) gene, in adult patients (25–65 years old) with early manifest Huntington's disease. The results showed that single or multiple doses of WVE‐120101 and WVE‐120102 were not associated with a significant reduction in mutant *HTT* levels compared to the placebo. Additionally, there was no strong evidence of a dose–response relationship at the tested doses. However, overall, the therapy was well tolerated, and most adverse events were of mild or moderate intensity including headache, procedure‐related pain, dizziness, back pain, falls, and upper respiratory viral infection.

Similarly, and with positive results, Phase 1 trials were conducted with WVE‐210201, another ASO designed to induce exon 51 skipping, in children aged 5–18 with Duchenne muscular dystrophy.^26^ This study concluded that the treatment is safe and well‐tolerated, with most adverse events being mild and manageable (i.e., diarrhea, nasopharyngitis). Although higher ASO doses presented more severe but temporary adverse events (e.g., cardiac disorder), the overall positive safety profile allowed progression to Phase 2/3 trials to further assess its efficacy and long‐term safety. However, in the Phase 2/3 trial, the treatment was withdrawn prematurely due to a lack of efficacy in increasing dystrophin expression as assessed by Western blot in muscle biopsies.^27^

Conversely, a placebo‐controlled, dose‐escalation study was conducted to evaluate the safety and biochemical efficacy of a morpholino oligonucleotide (AVI‐4658) that skips exon 51 in dystrophin mRNA in patients with Duchenne muscular dystrophy.^28^ Participants, boys aged 10–17 with Duchenne muscular dystrophy and deletions amenable to exon 51 skipping, received intramuscular injections of AVI‐4658. The study showed that the injection resulted in producing dystrophin correctly localized at the sarcolemma, with no adverse events or immune responses. In the low‐dose group, exon skipping was observed, but no significant dystrophin expression was detected. However, in the high‐dose group, strong dystrophin expression was observed, confirmed by immunohistochemistry and Western blot, suggesting that the produced protein would be functional and potentially clinically beneficial.

Other studies have yielded successful results like Nusinersen, which is an ASO designed to treat spinal muscular atrophy (SMA), a disease caused by mutations on chromosome 5q leading to survival motor neuron (SMN) protein deficiency. This drug increases the inclusion of exon 7 in *SMN2* mRNAs and the production of full‐length SMN proteins, enhancing the splicing efficiency of *SMN2* pre‐mRNA.^29^

These setbacks and positive results highlight the inherent challenges of this type of treatment and the complexity of developing effective therapies, although, at the same time, they offer hope for other diseases like DEEs. Results are still in the early stages as several clinical trials had to be stopped due to safety concerns or lack of efficacy of the drug.

One study has mainly focused on a large population of 60 pediatric participants with early onset *SCN2A*‐related DEE, aged 2–18 years old. The trial was aimed at determining the effect of the ASO PRAX‐222. Data are being collected and the estimated completion date for the primary phase is July 2025.^30^

The STK‐001 ASO, which promotes exon skipping in order to increase the amount of productive protein transcript, is also underway in the phase 1 clinical trial for the treatment of DS. The primary endpoint, as a phase 1 study, is to evaluate the safety and tolerability of single and multiple escalating doses of STK‐001 in patients IT injected. This is the first precision therapy for *SCN1A* gene‐linked DS to reach the clinical trials phase. STK‐001 is an ASO designed using the TANGO technology to increase *SCN1A* mRNA expression thus restoring the physiological levels of Na_V_1.1., which are decreased in people suffering from DS.^22^ For those patients who have completed the previous study of STK‐001 and meet study eligibility criteria, it was tested the safety of multiple doses of this ASO IT injected.^31^

A single case study involving an 18‐month‐old female diagnosed with a potassium channel mutation associated with epilepsy in infancy with migrating focal seizures was treated with an ASO therapy. Baseline sensory thresholds were recorded on the day of drug administration and they were compared with the predose and postdose sensory thresholds. The results showed that there was no statistical difference in predrug versus postdrug sensory thresholds, so no conclusive results have yet been reached.^32^ For more information see Table 3.

**TABLE 3:** Clinical studies of ASOs in DEEs.

Authors, year	Sample size	Study population	Methods	Type of developmental encephalopathies	Primary endpoints	Outcomes
NCT05737784	60	Pediatric	PRAX‐222 ascending doses	Early onset *SCN2A* DEE	Learn about the effect of PRAX‐222, preliminary safety, and dose escalation	Changes in seizure frequency and EEG‐based outcome measures
Cornelissen et al., 2022	1	Pediatric	IT injection with personalized ASO	Potassium channel mutation that provokes Infancy epilepsy with migrating focal seizures	Testing protocols, accounting for disease presentation, cognitive and motor function	Baseline sensory thresholds were recorded
NCT04442295	78	Two age groups: aged 13–18 years of age, inclusive, and from 2 to 12 years of age	Single and multiple ascending doses of STK‐001 administered as an IT injection	DS	Safety and tolerability of single and multiple doses of STK‐001, pharmacokinetic (PK) parameters, and exposure of STK‐001 in cerebrospinal fluid (CSF)	Change in seizure frequency, overall clinical status, and quality of life will be measured
NCT04740476	69	Two age groups: 13–18 years of age, inclusive, and 2–12 years of age who have completed the STK‐001 (NCT04442295) and meet study eligibility criteria	Multiple ascending doses of STK‐001 administered as an IT injection	DS	Safety of multiple doses of STK‐001	Change in seizure frequency and overall clinical status, and quality of life will be measured

Abbreviations: ASO, antisense oligonucleotide; DEE, developmental and epileptic encephalopathy; DS, Dravet syndrome; IT, intrathecal injection.

## 5 DISCUSSION

Although significant advances have been made in the chemical modification of oligonucleotides bringing the way to the advent of ASOs, their real therapeutic role is still largely unexplored. There are still significant challenges for ASO therapy in neurological disorders, due to their rapid degradation, the undesirable cellular uptake by cells that are not affected by the disease, the low penetrability linked to their high molecular weight and negative charge and, last but not least, the blood–brain barrier (BBB) that blocks the penetration of ASOs into the brain. Hence, currently, for CNS‐targeted therapies, ASOs must be administered by direct central injection.^33^

ASOs can be modified through a variety of chemical alterations, conferring greater resistance to nuclease degradation and better target affinity.^16^ In this way, the therapeutic properties of ASOs can be ameliorated and combined with the improvement given by novel routes of administration and biodistribution methods; for example, ASOs can be delivered into cells more efficiently using viral vectors, conjugated peptides, antibodies, and other nanoparticles (NPs) ligands or extracellular vesicles (EVs).^34^ Regarding animal models, since systemically administered ASOs do not cross the BBB, invasive techniques such as IT or ICV injection are required.^35^, ^36^ The most common human delivery methods in neurological disorders include intravitreal, intranasal, subcutaneous, intravenous, and the other more invasive ones (i.e., IT and ICV).^34^ Actually, none of the ASOs that have been approved by the FDA are delivered using particles, and they are all given to patients in their naked oligonucleotide form. There are ongoing clinical studies to assess the effectiveness of ASOs with delivery particles such as eplontersen,^37^ BP1001,^38^ vesleteplirsen,^39^ DYNE‐101,^40^ and DYNE‐251.^41^ Phase I studies demonstrated the safety of eplontersen and BP1001 administration, but these studies are still in their infancy.

Some trials are already implementing molecules that help to improve target range, and for this, ASO 3′ cholesterol tags have been used to increase cell absorption, although it has also been reported that this modification can potentially reduce the solubility of ASO.^4^ Also, metal NPs and functionalization of nucleic acid structures without restrictions, like multi‐layered coated gold nanoparticles (MLGNPs) that release ASO, were shown to be efficiently internalized into various species of gram‐positive bacteria and their use with antibiotics is also possible. These solutions have become extraordinarily promising for ASO delivery and personalized nanomedicine.^42^

*In vitro* models do not meet the minimum requirements for testing the safety of ASO therapies, and the selection of the *in vivo* model used must be carefully considered as safety criteria are not reliably extrapolated from one species to another. In addition, although no clear studies have been published presenting safety concerns, ASOs may remain in the body for a longer duration compared to other treatment modalities. The clearance period can be weeks or even months, therefore, ASO‐based antivenom strategies have been described, although these will require strict safety evaluations before they can be applied in the clinic. More data from human clinical trials are needed to assess the safety and clarify the true therapeutic potential of ASOs.^43^

Other recent studies of ASOs therapies designed to treat pediatric patients suffering from other neurological disorders are demonstrating that achieving treatment of DEEs with this strategy is not so far away. The treatment of SMA with ASOs from childhood gives more confidence in this approach and is very encouraging for patients with DS. Important questions remain to be addressed and cannot be answered using currently available mouse models.^17^ ASO studies have also been developed in the clinic for ultra‐rare and fatal neurodegenerative diseases, such as the launch of personalized therapy (the so‐called “n‐of‐1 trials”) for patients.

Other diseases such as Angelman syndrome (AS), a developmental disorder which may also present seizures, are being studied for therapies with ASOs.

AS is due to the absence of the maternal contribution of the imprinted region on chromosome 15q11‐q13 harboring the *UBE3A* causative gene. As such, the alteration of the maternal copy of *UBE3A* by different molecular events may not be compensated by the paternal allele which is subjected to imprinting and thus turned‐off. As a specific natural antisense transcript contributes to the silencing of the *UBE3A* paternal allele,^44^ an ASO‐based strategy has been designed to target this NAT and promote the expression of the gene from the paternal allele.^45^ To do this, an animal model of AS with the copy of the maternal gene blocked has been generated and injected with an ASO against paternal antisense transcript. After treatment, a recovery in protein levels was observed in both adult and young mice. EEG analysis also showed a partial recovery of the brain's electrical activity.^45^

More than 300 studies have been conducted on microRNAs (miRNAs) and epilepsy, mainly in animal models, but also in humans, in which the miRNA expression profile appears to be altered in the brain and blood of patients with different epileptic disorders. miRNAs are master modulators of gene expression and are involved in several neurodevelopmental processes, differentiation and maturation of neurons, and neuroinflammatory processes. As such, they play a functional role in epilepsy and may provide a therapeutic target. In this context, ASOs represent a promising tool for innovative therapies, with increasing evidence in preclinical models that the targeting of specific miRNAs leads to an amelioration of the epileptic phenotype. When the function of ASOs is to silence miRNAs, it is called antagomir. For example, a study showed that the ICV injection of an antagomir targeting miR‐134 in a kainic acid‐induced mouse model of epilepsy or a single intraperitoneal injection of the Anti‐miR134 in this model could potentially reduce the epileptic profile. Similarly, targeting miR‐146, miR‐34, miR‐135, miR‐324, miR‐10, miR‐21, miR‐199, miR‐203, mir‐210, miR‐132, and miR‐142 has shown a reduction in seizure frequency, with normalization of altered channel expression in some of these cases, while others, such as miR‐181, resulted in a recovery from neuronal apoptosis.^46^ Targeting miRNAs with antagomir has potent and long‐lasting anticonvulsant and even disease‐modifying effects in rodent models of drug‐resistant epilepsy and may promote the control of neuroinflammation, which is often altered in epilepsy.^46^

On the other hand, in some cases the expression level of specific miRNAs has to be preserved, restored, or increased and ASOs may be specifically designed to generate miRNA mimics called agomir. Indeed, studies with miR‐128 and miR‐124 agomirs reduced spontaneous seizures in mice.^46^

## 6 CONCLUSION

RNA‐based therapies have provided a shift in the way previously intractable genetic disorders are addressed, and the targeted approach of ASOs toward specific genetic anomalies in DEE is highly promising. ASO therapies have demonstrated favorable regulatory outcomes at cellular levels and, in preclinical models, have provided tangible enhancements in the clinical management of epileptic symptoms and cognitive development. However, there are still hurdles to overcome. A comprehensive exploration of ASO treatments for channel mutations and other genetic variations is imperative, with a paramount focus on establishing treatment safety and the assurance of sustained effectiveness through rigorous pharmacovigilance efforts. Notwithstanding, the swift‐growing body of research in this field gives hope for a successful future marked by ASO‐based therapies and personalized patient treatments.

## AUTHOR CONTRIBUTIONS

Paloma García Quilón carried out collection of data and drafting of the manuscript. Greta Volpedo, Serena Cappato, Loretta Ferrera, Federico Zara, and Renata Bocciardi carried out critical revision of the study. Antonella Riva and Pasquale Striano were involved in conception and design of the study, revision, and final approval of the manuscript. All authors agree to be accountable for all aspects of the work.

## FUNDING INFORMATION

No targeted funding to be reported.

## CONFLICT OF INTEREST STATEMENT

Neither of the authors has any conflict of interest to disclose.

## PATIENT CONSENT STATEMENT

Not applicable as no human subjects were involved in the study.

## Data Availability

Data sharing is not applicable to this article as no datasets were generated or analyzed during the current study.
